# Electroosmotically actuated peristaltic-ciliary flow of propylene glycol + water conveying titania nanoparticles

**DOI:** 10.1038/s41598-023-38820-4

**Published:** 2023-07-21

**Authors:** Javaria Akram, Noreen Sher Akbar

**Affiliations:** 1 School of Natural Sciences (SNS), National University of Sciences and Technology (NUST), Islamabad, 44000 Pakistan; 2 DBS&H, CEME, National University of Sciences and Technology, Islamabad, Pakistan

**Keywords:** Mathematics and computing, Nanoscience and technology

## Abstract

The main focus of this article is to mathematically formulate the microfluidics-based mechanical system for nanofluids. A 50:50 mixture of propylene glycol (PG) and water is used as a heat transfer fluid because of its tremendous anti-freezing properties, and nontoxicity and it is safe to be utilized at the domestic level. Titanium dioxide (titania) nanoparticles are suspended in the working fluid to enhance its heat transfer ability. The fluid flow is induced by electroosmosis in a microtube, which is further assisted by cilia beating. The impacts of Joule heating and non-linear thermal radiation are also considered. The simplification of the dimensionless system is done under lubrication theory and the Debye-Hückel linearization principle. The nonlinear system of equations is executed for a numerical solution by adopting the symbolic mathematical software Maple 17 using the command “dsolve” along with the additional command “numeric” to get the numerical solution. This command utilizes a low-ordered method along with accuracy-enhancing schemes such as the deferred correction technique and Richardson extrapolation to get a numerical answer of desired accuracy, where we can choose the accuracy level and mesh points according to our requirements. The detailed analysis of results obtained from the numerical treatment of the considered problem indicates that the efficiency of the PG + water enhances due to the suspension of the nanoparticles and heat is rapidly removed from the system. Further, the velocity of the fluid is augmented by decreasing the thickness of the electric double layer and raising the strength of the electric field in the forwarding direction.

## Introduction

Most substances acquire a net surface charge when they come across an aqueous polar medium. Due to the interaction between a charged surface and ionic species, the diffuse layer is generated. The ions in the diffuse layer experience an axial body force in the presence of an external force. Such a kind of motion is termed as electroosmosis which is considered as a very suitable mechanism for transportation purposes in microfluidic devices. In recent years, microfluidic devices have been triggered by the growing needs of biomedical engineering as well as energy systems. For the fabrication of microfluidics devices, there is a need to develop theoretical as well as experimental models of electroosmotic flow with heat transfer assisted by the various pumping mechanisms. Xuan et al.^1^ have presented a basic model of electroosmotic flow where they have discussed the joule heating effects. Recently, Khan and Sasmal^2^ investigated the electroosmotic flow phenomenon for both Newtonian and viscoelastic fluids through a porous environment containing long micropores. An investigation on the coupled impact of pressure-driven flow and electrokinetics is carried out by Yuan et al.^3^ where they did not ignore the adverse pressure impact on electroosmotic flow. A mathematical model for electroosmotic transport of two immiscible fluids is developed and analyzed by Alyousef et al.^4^ in which they utilized the Ellis fluid model for the viscoelastic behavior of the considered fluid. Cilia beating is a natural mechanism of physiology that generates effective stroke and recovery stroke. Due to an effective stroke, water/fluid moves backward and cilia move forward. Due to recovery strokes, cilia further come back to their original position. These effective recovery strokes are rhythmic processes and generate metachronal waves. Cilia beating is a key mechanism of the respiratory system that cleans dust particles. In some physiological systems, cilia work as sensors and actuators. Some of the basic studies^5–9^ on cilia transport and its roles have been reported. From a natural perspective, it is an essential mechanism of the physiological system. Based on the extensive role of this mechanism, nowadays, there are challenging demands for artificial cilia and artificial magnetic cilia for the sensors and actuators^10^, for rotating machines to compute the frequency^11^, for fluid pumping^12^, and many more. Inspired by the natural and artificial roles of the cilia beating in fluid dynamics and biomedical sciences, most recently researchers have developed some mathematical models on the cilia-assisted rheological fluid flow^13–19^ and MHD fluid flow^20–23^ where they have discussed the effects of key parameters such as cilia length, the eccentricity of the metachronal waves, curvature of the flow geometry, magnetic field and rheological parameters on fluid pumping. In order to enhance the efficiency of microfluidic devices, different flow-causing mechanisms can be combined to achieve improved flow and heat transfer rates. The electroosmotic flows, triggered by ciliary motion can be helpful in the development of electromechanical devices at the microscale such as in micro and nanofluidic pumps, hemodialysis, cooling chips, microfabricated liquid devices, etc. Owing to the stunning efficiency of combined ciliary-actuated electroosmotic flows, recently few researchers have put forward their efforts in investigating the properties of flow driven by the combined phenomenon. Abdelsalam and Zaher^24^ studied the impact of electroosmotic forces on the movement of sperm in a ciliated cervical canal in which they employed the hyperbolic-tangent fluid to model the cervical fluid. Imran et al.^25^ investigated the heat transfer properties of Williamson fluid flow where the fluid flow is generated by electroosmosis and cilia beating in the micro-ciliated channel. Some more significant studies are reported by^26^, Ijaz et al.^27^, and Javed et al.^28^.

Solar thermal technology consists of setups that collect the energy from the sun and convert it into heat energy. This heat energy is then stored by using water as a working fluid and utilized in various applications. The components of a solar heating system contain a source for collecting the solar energy, i.e. collector, a means of exchanging the heat using the flow of the fluid, namely the heat transfer circuit, the heat transferring fluid, and an energy storage system. In a climate, where there occur extreme temperature conditions in different parts of the year, the usage of anti-boiling and anti-freezing fluids is recommended in order to avoid damage to the solar system due to corrosion, overheating, and freezing. For this purpose, multiple types of anti-freezing agents are present, such as ethylene glycol (EG), tri-ethylene glycol, propylene glycol (PG), etc., which have the capability of protecting the solar system from freezing and rust. But these antifreeze agents in their pure form do not possess good thermal properties, which is the main requirement of a solar system. Therefore, a mixture of these anti-freezing liquids with water is utilized for this purpose. Out of different antifreeze/water solutions, the most commonly used solution is propylene glycol/water solution. Due to its low cost, high performance, low corrosivity, least toxicity, and environmentally friendly nature, as found by Heinonen et al.^29^ in a comparative study, it is the most suitable anti-freezing fluid, especially for use in domestic-level devices. Shojaeizadeh et al.^30^ performed an experiment to investigate the performance of a flat plate solar cell using different concentrations of PG in water. They observed that upon increasing the concentration of PG in water from 25 to 75%, an improvement in the efficiency of the solar cell occurs. Jugar and Crook^31^ compared the performance of PG and EG with their different concentrations in water and concluded that it is necessary to maintain a 50/50 concentration of water and antifreeze in order to keep a balance between heat transfer properties and antifreeze protection.

Titanium dioxide (TiO_2_), which is one of the oxides of titanium, was first discovered from ilmenite in 1791. Due to its high refractive index, and low chances of decolorization, it is mainly used in pigments^32^. However, other than its large-scale usage in pigments, TiO_2_ is also used in enamels, water purification, cosmetics, food items^33^, and in energy storage devices. Titanium dioxide is most beneficial when utilized in the form of small particles (usually nanosized) due to its enhanced light absorption tendency, photocatalytic reduction, and high surface photoactivity. Titanium dioxide nanomaterials are employed as photocatalysts in water purification as they can effectively degrade the organic and inorganic pollutants present in wastewater^34,35^. Due to the semiconducting nature of titanium dioxide nanoparticles, they are widely utilized in electronic components, catalytical electrodes, and solar cells. Titanium dioxide nanoparticles are very preferable to be used in domestic-level devices due to their non-toxic nature^36^ and easy and cost-effective availability.

The heat transfer mechanism offered by ordinary fluids such as ethylene glycol, water, engine oils, etc. is highly inefficient due to their innate poor conductivity. As already mentioned the heat transfer process is one of the major factors affecting the efficiency of solar cells but the addition of antifreeze in water lowers its heat transfer ability. In order to overcome the demands of energy-efficient devices in this modern era, a new class of fluid offering potentially improved heat transfer rates is required. It was Choi^37^, who contributed his efforts to the generation of a new class of fluids named as “nanofluids” offering exciting applications in improving the heat transfer ability of the fluids by the insertion of nanosized particles (1 nm–100 nm) in the conventional heat transfer fluids. The extraordinarily high heat transfer ability of these new-generation fluids triggers their usage in pharmaceuticals, nano-scaled lubrication in rocket engines, cooling/heating of electronic devices, vehicle thermal management, solar cells, and many more. Such promising applications of nanofluids attracted the fluid dynamists to investigate such kinds of fluid and even more innovative fluids having more than one type of solid nanoparticles which are usually named as “hybrid nanofluids”. In an experimental study performed by Javidan and Moghadam^38^ an effective cooling for photovoltaic cells is tested by using SiC/water nanofluid as a cooling agent. Saleem et al.^39^ investigated the importance of thermophoresis and Brownian motion on the motion of water consisting of three different types of nanoparticles. Elnaqeeb et al.^40^ presented the importance of dual stretching and suction in the flow of water-based ternary-hybrid nanofluids by assuming multiple shapes and densities of nanoparticles. Animasaun et al.^41^ studied the motion of water, containing carbon nanotubes, copper, and graphene nanoparticles of different shapes, over stagnant moveable walls. They concluded that a minimum value of local skin friction coefficient can be achieved when a larger fraction of nanoparticles are used. Many other investigations focusing on the applications of multiple types of nanofluids are contributed by Rasool et al.^42^, Adnan and Ashraf^43^, Xiu et al.^44^, and Shah et al.^45^.

Due to the above-mentioned advantages of the propylene-glycol/water mixture and titanium dioxide nanoparticles, a combination of these two as a nanofluid can be adapted to work as heat transfer fluid in domestic-level devices. Propylene-glycol/water-based TiO_2_ nanofluid can provide a sufficient rate of heat transfer along with the most important constraint of non-toxicity. So the aim here is to investigate the heat and mass transfer capability of propylene-glycol/water-based TiO_2_ nanofluid which is driven by the combined influence of cilia beating and the electroosmotic forces. Nevertheless, none of the studies have focused on the mathematical model of the cilia-assisted electroosmotic flow of nanofluids which could be applicable in various biomedical and energy fields. Based on research gaps, herein, a mathematical model is presented to study the propylene glycol + water-based TiO_2_ nanofluids flow driven by the electroosmosis and cilia beating in a microtube. The present results suggest that this model can be applied to the household solar system. This model is structured in the form of an introduction followed by mathematical formulation and numerical solutions. Thereafter, a thorough discussion of simulated results has been presented. In last, the concluding remarks of the present analysis have been listed.

## Mathematical formulation

### Ciliary movement

**Figure 1:**
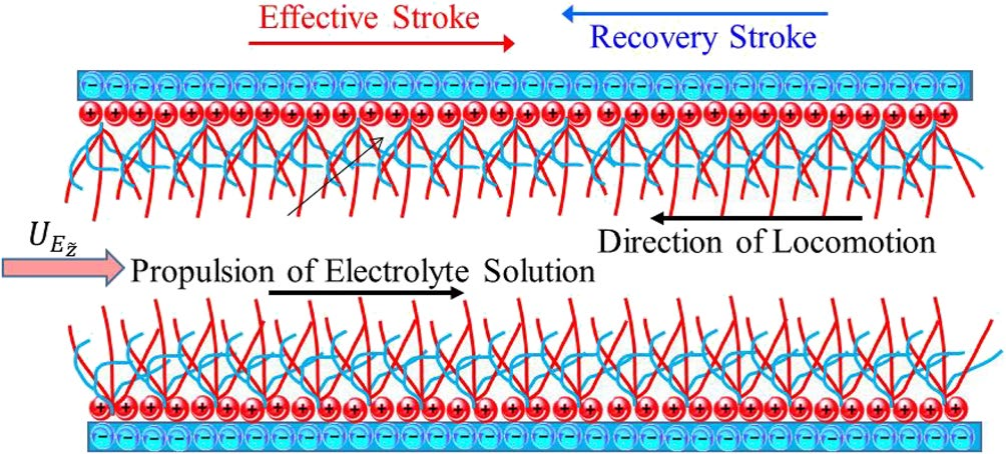
Schematic representation of the cilia-assisted electroosmotic flow.

Here we have considered the characteristics of fluid motion of 50:50 ionic water-propylene glycol (PG) solution-based titanium dioxide nanoparticles through a ciliated tube. A metachronal wave produced by the cumulative beating of cilia travels with the speed *c* towards the right (see Fig. 1). Further, the electroosmotic motion in the fluid is generated by the implementation of an external electric field across the tube in the z-direction. The envelope for the elliptical path followed by cilia tips is represented mathematically by choosing the polar coordinate system ($\tilde{r},\tilde{z},\tilde{t}$) and given as^46^:

1 $$\tilde{r} = \tilde{m}\left( {\tilde{z},\tilde{t}} \right) = d + d\epsilon_{1} {\text{cos}}\left( {\frac{2\pi }{\eta }\left( {\tilde{z} - c\tilde{t}} \right)} \right),$$

2 $$\tilde{z} = \tilde{n}\left( {\tilde{z}_{0} ,\tilde{z},\tilde{t}} \right) = \tilde{z}_{0} + d\epsilon_{1} \tilde{\alpha }{\text{sin}}\left( {\frac{2\pi }{\eta }\left( {\tilde{z} - c\tilde{t}} \right)} \right),$$

in which d designates the tube radius, $\epsilon_{1}$ the dimensionless length of the cilia, $\tilde{z}_{0}$ the cilia reference point and $\tilde{\alpha }$ the eccentricity for the elliptic path followed by cilia.

Imposing no-slip conditions across the tube walls, the velocity of the fluid adjacent to the cilia is the same as the velocity of the cilia, therefore, the axial $\tilde{w}$ and radial velocity $\tilde{u}$ of the cilia at the reference point can be expressed as:

3 $$\tilde{w} = \left. {\frac{{\partial \tilde{z}}}{{\partial \tilde{t}}}} \right|_{{z = \tilde{z}_{0} }} = \frac{{\partial \tilde{n}}}{{\partial \tilde{t}}} + \frac{{\partial \tilde{n}}}{{\partial \tilde{z}}}\frac{{\partial \tilde{z}}}{{\partial \tilde{t}}} = \frac{{\partial \tilde{n}}}{{\partial \tilde{t}}} + \frac{{\partial \tilde{n}}}{{\partial \tilde{z}}}\tilde{w},$$

4 $$\tilde{u} = \left. {\frac{{\partial \tilde{r}}}{{\partial \tilde{t}}}} \right|_{{z = \tilde{z}_{0} }} = \frac{{\partial \tilde{m}}}{{\partial \tilde{t}}} + \frac{{\partial \tilde{m}}}{{\partial \tilde{z}}}\frac{{\partial \tilde{z}}}{{\partial \tilde{t}}} = \frac{{\partial \tilde{m}}}{{\partial \tilde{t}}} + \frac{{\partial \tilde{m}}}{{\partial \tilde{z}}}\tilde{w}.$$

Using the relations given in Eqs. (1)–(2) in Eqs. (3)–(4) result in the following expressions:

5 $$\tilde{w}\left( {\tilde{z},\tilde{t}} \right) = - \frac{{\frac{2\pi }{\eta }dc\epsilon_{1} \tilde{\alpha }{\text{cos}}\left( {\frac{2\pi }{\eta }\left( {\tilde{z} - c\tilde{t}} \right)} \right)}}{{1 - \frac{2\pi }{\eta }d\epsilon_{1} \tilde{\alpha }{\text{cos}}\left( {\frac{2\pi }{\eta }\left( {\tilde{z} - c\tilde{t}} \right)} \right)}},$$

6 $$\tilde{u}\left( {\tilde{z},\tilde{t}} \right) = \frac{{\frac{2\pi }{\eta }dc\epsilon_{1} {\text{sin}}\left( {\frac{2\pi }{\eta }\left( {\tilde{z} - c\tilde{t}} \right)} \right)}}{{1 - \frac{2\pi }{\eta }d\epsilon_{1} \tilde{\alpha }{\text{cos}}\left( {\frac{2\pi }{\eta }\left( {\tilde{z} - c\tilde{t}} \right)} \right)}},$$

### Governing equations

This analysis focuses on the flow phenomenon of PG/water solution-based titanium dioxide (TiO_2_) nanoparticles driven by the combined metachronal wave and the electroosmotic body forces. The considered nanofluid exhibits the Newtonian characteristics which are governed through the Navier–Stokes equation. The conservation equations for the nanofluid are formulated by employing the Buongiorno flow model along with the relation of thermal conductivity and viscosity proposed by Corcione’s model. The electric potential distribution within the fluid medium is governed by the linearized Poisson-Boltzmann equation and simplified under the Debye-Hückel approximation of low zeta potential. The heat transfer analysis is performed in the presence of nonlinear thermal radiation and Joule heating. The slip conditions for temperature and zero mass flux for the nanoparticle volume fraction are applied across tube walls.

Subject to above defined physical conditions, the constitutive equations for momentum, energy, and concentration equations are formulated as:

7 $$\frac{{\partial \tilde{u}}}{{\partial \tilde{r}}} + \frac{{\tilde{u}}}{{\tilde{r}}} + \frac{{\partial \tilde{w}}}{{\partial \tilde{z}}} = 0,$$

8 $$\rho_{nf} \left( {\frac{{\partial \tilde{u}}}{{\partial \tilde{t}}} + \tilde{u}\frac{{\partial \tilde{u}}}{{\partial \tilde{r}}} + \tilde{w}\frac{{\partial \tilde{u}}}{{\partial \tilde{z}}}} \right) = - \frac{{\partial \tilde{p}}}{{\partial \tilde{r}}} + \mu_{nf} \left( {\frac{{\partial^{2} \tilde{u}}}{{\partial \tilde{r}^{2} }} + \frac{1}{{\tilde{r}}}\frac{{\partial \tilde{u}}}{{\partial \tilde{r}}} - \frac{{\tilde{u}}}{{\tilde{r}^{2} }} + \frac{{\partial^{2} \tilde{u}}}{{\partial \tilde{z}^{2} }}} \right) + \rho_{e} U_{{E_{{\tilde{r}}} }} ,$$

9 $$\begin{aligned} \rho_{nf} \left( {\frac{{\partial \tilde{w}}}{{\partial \tilde{t}}} + \tilde{u}\frac{{\partial \tilde{w}}}{{\partial \tilde{r}}} + \tilde{w}\frac{{\partial \tilde{w}}}{{\partial \tilde{z}}}} \right) & = - \frac{{\partial \tilde{p}}}{{\partial \tilde{z}}} + \mu_{nf} \left( {\frac{{\partial^{2} \tilde{w}}}{{\partial \tilde{r}^{2} }} + \frac{1}{{\tilde{r}}}\frac{{\partial \tilde{w}}}{{\partial \tilde{r}}} + \frac{{\partial^{2} \tilde{w}}}{{\partial \tilde{z}^{2} }}} \right) + \rho_{e} U_{{E_{{\tilde{z}}} }} \\ & \;\;\;\; + \left( {\rho \gamma } \right)_{nf} g\left( {\tilde{T} - T_{0} } \right) + \rho_{nf} g\beta_{{\tilde{\Phi }}} \left( {\tilde{\Phi } - \Phi_{0} } \right), \\ \end{aligned}$$

10 $$\begin{aligned} \left( {\rho C} \right)_{nf} \left( {\frac{{\partial \tilde{T}}}{{\partial \tilde{t}}} + \tilde{u}\frac{{\partial \tilde{T}}}{{\partial \tilde{r}}} + \tilde{w}\frac{{\partial \tilde{T}}}{{\partial \tilde{z}}}} \right) & = K_{nf} \left( {\frac{{\partial^{2} \tilde{T}}}{{\partial \tilde{r}^{2} }} + \frac{1}{{\tilde{r}}}\frac{{\partial \tilde{T}}}{{\partial \tilde{r}}} + \frac{{\partial^{2} \tilde{T}}}{{\partial \tilde{z}^{2} }}} \right) - \frac{{\partial q_{{\tilde{r}}} }}{{\partial \tilde{r}}} + \sigma_{nf} U_{{E_{{\tilde{z}}} }}^{2} \\ & \;\;\;\; + \left( {\rho C} \right)_{s} D_{B} \left( {\frac{{\partial \tilde{\Phi }}}{{\partial \tilde{r}}}\frac{{\partial \tilde{T}}}{{\partial \tilde{r}}} + \frac{{\partial \tilde{\Phi }}}{{\partial \tilde{z}}}\frac{{\partial \tilde{T}}}{{\partial \tilde{z}}}} \right) + \frac{{D_{{{T}}} }}{{T_{m} }}\left( {\rho C} \right)_{s} \left( {\left( {\frac{{\partial \tilde{T}}}{{\partial \tilde{r}}}} \right)^{2} + \left( {\frac{{\partial \tilde{T}}}{{\partial \tilde{z}}}} \right)^{2} } \right), \\ \end{aligned}$$

11 $$\begin{aligned} \left( {\frac{{\partial \tilde{\Phi }}}{{\partial \tilde{t}}} + \tilde{u}\frac{{\partial \tilde{\Phi }}}{{\partial \tilde{r}}} + \tilde{w}\frac{{\partial \tilde{\Phi }}}{{\partial \tilde{z}}}} \right) & = D_{B} \left( {\frac{{\partial^{2} \tilde{\Phi }}}{{\partial \tilde{r}^{2} }} + \frac{1}{{\tilde{r}}}\frac{{\partial \tilde{\Phi }}}{{\partial \tilde{r}}} + \frac{{\partial^{2} \tilde{\Phi }}}{{\partial \tilde{z}^{2} }}} \right) \\ & \;\;\;\; + \frac{{D_{T} }}{{{T}_{m} }}\left( {\frac{{\partial^{2} \tilde{T}}}{{\partial \tilde{r}^{2} }} + \frac{1}{{\tilde{r}}}\frac{{\partial \tilde{T}}}{{\partial \tilde{r}}} + \frac{{\partial^{2} \tilde{T}}}{{\partial \tilde{z}^{2} }}} \right). \\ \end{aligned}$$

The nonlinear radiative heat flux computed by Roseland’s approximation is given by:

12 $$\begin{aligned} q_{r} & = - \frac{{4\sigma^{*} }}{{3\kappa^{*} }}\frac{{\partial \tilde{T}^{4} }}{{\partial \tilde{r}}}, \\ q_{r} & = - \frac{{16\sigma^{*} }}{{3\kappa^{*} }}\tilde{T}^{3} \frac{{\partial \tilde{T}}}{{\partial \tilde{r}}}. \\ \end{aligned}$$

where $\sigma^{*}$ and $\kappa^{*}$ symbolize the Stefan-Boltzmann constant and mean absorption coefficient respectively.

In the above equations, $\tilde{p}$ stands for pressure, $\rho_{nf}$ for the nanofluid density, $\rho_{e}$ for the electric current density, $U_{{E_{{\tilde{r}}} }}$ and $U_{{E_{{\tilde{z}}} }}$ for the radial and the axial electric forces, $\left( {\rho \gamma } \right)_{nf}$ for the thermal expansion coefficient, $\beta_{{\tilde{\Phi }}}$ for the coefficient of mass expansion, $\tilde{T}$ and $\tilde{\Phi }$ for temperature and the nanoparticle volume fraction respectively, $\sigma_{nf}$ for the nanofluid electric conductivity, $D_{T}$ and $D_{B}$ for the coefficient of thermophoretic and diffusion motion respectively and $\left( {\rho C} \right)_{nf}$ for the specific heat capacity of nanofluid.

The relations for nanofluid properties are expressed as^40^:

13 $$\rho_{nf} = \left( {1 - \Phi_{0} } \right)\rho_{bf} + \Phi_{0} \rho_{p} ,$$

14 $$\begin{aligned} \left( {\rho C} \right)_{nf} & = \left( {1 - \Phi_{0} } \right)\left( {\rho C} \right)_{bf} + \Phi_{0} \left( {\rho C} \right)_{p} , \\ \left( {\rho \gamma } \right)_{nf} & = \left( {1 - \Phi_{0} } \right)\left( {\rho \gamma } \right)_{bf} + \Phi_{0} \left( {\rho \gamma } \right)_{p} , \\ \end{aligned}$$

with $\rho_{bf}$ and $\rho_{p}$ representing the densities of the base fluid and titanium oxide nanoparticles and $\Phi_{0}$ the fraction of nanoparticles being scattered in the base fluid.

Corcione’s model for thermal conductivity is given by^47^:

15 $$\frac{{ k_{nf} }}{{k_{bf} }} = \left( {1 + 4.4R^{0.4} Pr^{0.66} \left( {\frac{{T_{0} }}{{T_{fr} }}} \right)^{10} \left( {\frac{{k_{p} }}{{k_{bf} }}} \right)^{0.03} \Phi_{0}^{0.66} } \right) = k_{r} ,$$

16 $$\frac{{ \mu_{nf} }}{{\mu_{bf} }} = \frac{1}{{\left( {1 - 34.87\left( {\frac{{d_{p} }}{{d_{bf} }}} \right)^{ - 0.3} \Phi_{0}^{1.03} } \right)}} = \mu_{r} ,$$

In the above relations, $T_{fr}$ specifies the freezing temperature of the base fluid, $R$ the nanoparticles’ Reynolds number, Pr the Prandtl number and $d_{bf}$ and $d_{p}$ the diameters of base fluid molecules and nanoparticles respectively.

The Maxwell–Garnett model for electrical conductivity is expressed as^48^:

17 $$\frac{{\sigma_{nf} }}{{\sigma_{bf} }} = \left( {1 + \frac{{\left( {\frac{{\sigma_{p} }}{{\sigma_{bf} }} - 1} \right)\Phi_{0} }}{{\left( {\frac{{\sigma_{p} }}{{\sigma_{bf} }} + 2} \right) - \left( {\frac{{\sigma_{p} }}{{\sigma_{bf} }} - 1} \right)\Phi_{0} }}} \right) = \sigma_{r} .$$

The Poisson equation for electric potential distribution in the fluid medium, due to by motion of ions generated by the external electric field, derived in polar coordinates is given by:

18 $$\frac{{\partial^{2} U_{{\tilde{E}}} }}{{\partial \tilde{r}^{2} }} + \frac{1}{{\tilde{r}}}\frac{{\partial U_{{\tilde{E}}} }}{{\partial \tilde{r}}} + \frac{{\partial^{2} U_{{\tilde{E}}} }}{{\partial \tilde{z}^{2} }} = - \frac{{\rho_{e} }}{{\varepsilon_{0} \varepsilon_{r} }},$$

where ε_r_ and $\varepsilon_{0}$ designate the relative permittivity for the base fluid and the permittivity of vacuum respectively.

The electric charge density in terms of the number densities of anions $n^{ - }$ and cations $n^{ + }$ having valency $z$ is defined as:

19 $$\rho_{e} = ez\left( {n^{ + } - n^{ - } } \right){ }$$

Defining the transformation relations between a fixed frame of reference and wave frame

20 $$\begin{aligned} z^{*} & = \tilde{z} - c\tilde{t}, r^{*} = \tilde{r}, w^{*} = \tilde{w} - c, \\ u^{*} & = \tilde{u}, p^{*} \left( {r^{*} ,z^{*} } \right) = \tilde{p}\left( {\tilde{r},\tilde{z},\tilde{t}} \right). \\ \end{aligned}$$

### Scaling analysis

The following dimensionless parameters are defined as:

21 $$\begin{aligned} r & = \frac{{r^{*} }}{d}, z = \frac{{ z^{*} }}{\eta }, p = \frac{{ p^{*} d^{2} }}{{\mu_{bf} c\eta }}, n = \frac{{\tilde{n}}}{{n_{0} }},A_{1} = \frac{{\rho_{nf} }}{{\rho_{bf} }},h = 1 + \epsilon_{1} {\text{cos}}\left( {2\pi z} \right), \\ \delta & = \frac{d}{\eta }, \theta = \frac{{\tilde{T} - T_{0} }}{{(T_{1} - T_{0} )}},{\text{Gr}}_{t} = \frac{{\rho_{bf} g\gamma_{bf} d^{2} (T_{1} - T_{0} )}}{{\mu_{bf} c}}, {\text{S}} = \frac{{\sigma_{bf} U_{{E_{z} }}^{2} d^{2} }}{{k_{bf} (T_{1} - T_{0} )}}, \\ \Phi & = \frac{{\tilde{\Phi } - \Phi_{0} }}{{\Phi_{0} }} ,U_{E} = \frac{{ezU_{{\tilde{E}}} }}{{k_{B} \hat{T}_{avg} }},{\text{Re}} = \frac{{\rho_{bf} cd}}{{\mu_{bf} }} , \tau^{*} = \frac{{\left( {\rho c} \right)_{p} }}{{\left( {\rho c} \right)_{bf} }}, {\text{Pr}} = \frac{{\mu_{bf} C_{bf} }}{{k_{bf} }} \\ U_{0} & = - \frac{{\varepsilon_{0} \varepsilon_{r} k_{B} \hat{T}_{avg} U_{{E_{z} }} }}{{ez\mu_{bf} c}}, k = \sqrt {\frac{{2n_{0} e^{2} z^{2} d^{2} }}{{\varepsilon_{0} \varepsilon_{r} k_{B} \hat{T}_{avg} }}} = \frac{d}{{\lambda_{d} }}, N_{t} = \frac{{D_{{{T}}} \tau^{*} \rho_{bf} (T_{1} - T_{0} )}}{{T_{m} \mu_{bf} }} , \\ N_{b} & = \frac{{D_{B} \tau^{*} \rho_{bf} \Phi_{0} }}{{\mu_{bf} }}, L = \frac{{\left( {\rho \gamma } \right)_{nf} }}{{\left( {\rho \gamma } \right)_{bf} }},{\text{Gr}} = \frac{{\rho_{bf} g\beta_{{\overline{\Phi }}} d^{2} \Phi_{0} }}{{\mu_{bf} c}}, R_{d} = \frac{{16\sigma^{*} T_{0}^{3} }}{{3k^{*} \mu_{bf} C_{bf} }}, \\ u & = - \frac{\delta }{r}\Psi_{z} , w = \frac{1}{r}\Psi_{r} , \eta_{w} = \frac{{T_{1} }}{{T_{0} }}, \Psi = \frac{{\tilde{\Psi }}}{cd}. \\ \end{aligned}$$

In which, ${\text{Re}}$ designates the Reynolds number, $U_{0}$ the electroosmotic velocity parameter, $k$ the Debye length parameter, $N_{t}$ the dimensionless thermophoretic parameter, ${\text{Gr}}_{t}$ the thermal Grashof number, $N_{b}$ the Brownian motion parameter, $R_{d}$ the radiation parameter, $S$ the Joule heating parameter, ${\text{Pr}}$ the Prandtl number, $\theta$ the dimensionless parameter, ${\text{Gr}}_{\Phi }$ the mass transfer Grashof number, $\delta$ the wavenumber, $\eta_{w}$ the temperature ratio parameter, $\Psi$ the stream function, and $\Phi$ the dimensionless nano fraction parameter.

Using Eqs. (20) and (21) in the dimensionless analysis of Eqs. (7)–(11) and (18)–(19) and adopting the assumption of small wavenumber and the domination of viscous forces over inertial forces, we get the following simplified set of equations,

22 $$\frac{\partial p}{{\partial r}} = 0,$$

23 $$\frac{\partial p}{{\partial z}} = \frac{{\mu_{r} }}{r}\frac{{\partial \left( {rw} \right)}}{\partial r} + \frac{{U_{0} }}{r}\frac{\partial }{\partial r}\left( {r\frac{{\partial U_{E} }}{\partial r}} \right) + {\text{Gr}}_{t} L\theta + {\text{Gr}}_{\Phi } A_{1} \Phi ,$$

24 $$0 = \frac{\partial }{\partial r}\left( {\frac{{\mu_{r} }}{r}\frac{{\partial \left( {rw} \right)}}{\partial r}} \right) + \frac{{U_{0} }}{r}\frac{{\partial^{2} }}{{\partial r^{2} }}\left( {r\frac{{\partial U_{E} }}{\partial r}} \right) + {\text{Gr}}_{t} L\frac{\partial \theta }{{\partial r}} + {\text{Gr}}_{\Phi } A_{1} \frac{\partial \Phi }{{\partial r}},$$

25 $$\begin{aligned} \left( {\frac{{k_{nf} }}{{k_{bf} }}} \right)\left( {\frac{{\partial^{2} \theta }}{{\partial r^{2} }} + \frac{1}{r}\frac{\partial \theta }{{\partial r}}} \right) & + PrN_{b} \frac{\partial \theta }{{\partial r}}\frac{\partial \Phi }{{\partial r}} + PrN_{t} \left( {\frac{\partial \theta }{{\partial r}}} \right)^{2} + R_{d} \frac{{\partial^{2} \theta }}{{\partial r^{2} }}\left( {\theta \left( {\eta_{w} - 1} \right) + 1} \right)^{3} \\ & + 3R_{d} \left( {\theta \left( {\eta_{w} - 1} \right) + 1} \right)^{2} \left( {\eta_{w} - 1} \right)\left( {\frac{\partial \theta }{{\partial r}}} \right)^{2} + \sigma_{r} S = 0, \\ \end{aligned}$$

26 $$\frac{{\partial^{2} \Phi }}{{\partial r^{2} }} + \frac{1}{r}\frac{\partial \Phi }{{\partial r}} + \frac{{N_{t} }}{{N_{b} }}\left( {\frac{{\partial^{2} \theta }}{{\partial r^{2} }} + \frac{1}{r}\frac{\partial \theta }{{\partial r}}} \right) = 0,$$

27 $$\frac{1}{r}\frac{\partial }{\partial r}\left( {r\frac{{\partial U_{E} }}{\partial r}} \right) = k^{2} \left( {\frac{{n^{ - } - n^{ + } }}{2}} \right).$$

The Boltzmann distribution for the local ionic density of each ionic species is defined as:

28 $$n^{ \pm } = e^{{ \mp U_{E} }} = e^{{ \mp \frac{{ezU_{E} }}{{k_{B} \hat{T}_{avg}}}}} ,$$

Substituting the Boltzmann relations in Eq. (27), we get,

29 $$\frac{1}{r}\frac{\partial }{\partial r}\left( {r\frac{{\partial U_{E} }}{\partial r}} \right) = k^{2} \sinh \left( {U_{E} } \right),$$

In order to linearize Eq. (29), we can make use of Debye–Hückel linearization principle which is basically assumption of smaller zeta potential in the diffuse layer. Use of this linearization principle is quite justified as for a wide range of PH of electrolyte solution, zeta potenetial remains lower than 25 mV so we can make use of assumption $\sinh \left( {U_{E} } \right) \approx U_{E}$ in Eq. (29) which reduces to

30 $$\frac{1}{r}\frac{\partial }{\partial r}\left( {r\frac{{\partial U_{E} }}{\partial r}} \right) = k^{2} U_{E} ,$$

Subject to the appropriate boundary conditions defined below

31 $$U_{E} |_{r = h} = \xi , U_{E} |_{r = 0} = 0,$$

Equation (30) can be solved directly to get an analytical expression for the potential field as:

32 $$U_{E} = \xi \frac{{I_{0} \left( {kr} \right)}}{{I_{0} \left( {kh} \right)}},$$

Here $I_{0}$ represents the modified Bessel functions of 1^st^ kind.

The suitable boundary conditions in the dimensionless form are prescribed as:

33 $$\begin{aligned} \psi & = 0, - \frac{1}{{r^{2} }}\frac{\partial \psi }{{\partial r}} + \frac{1}{r}\frac{{\partial^{2} \psi }}{{\partial r^{2} }} = 0,\frac{\partial \theta }{{\partial r}} = 0,\frac{\partial \Phi }{{\partial r}} + \frac{{N_{t} }}{{N_{b} }}\frac{\partial \theta }{{\partial r}} = 0, \;\;\;\;{\text{at}}\; r = 0, \\ \psi & = F,\frac{1}{r}\frac{\partial \psi }{{\partial r}} = - 1 - 2\pi \delta \epsilon_{1} d{\text{cos}}\left( {2\pi z} \right),\theta + \beta \frac{\partial \theta }{{\partial r}} = 0, \frac{\partial \Phi }{{\partial r}} + \frac{{N_{t} }}{{N_{b} }}\frac{\partial \theta }{{\partial r}} = 0,\;\;\;\; {\text{at}}\; r = h, \\ \end{aligned}$$

where $\beta$ represents the temperature slip parameter.

The instantaneous volume flow rate in the laboratory frame is given as

34 $$\mathop Q\limits^{\prime } = \mathop \smallint \limits_{0}^{h} \tilde{r}\tilde{w}d\tilde{r},$$

35 $$\mathop Q\limits^{\prime } = \mathop \smallint \limits_{0}^{h} r^{*} \left( {w^{*} + c} \right)dr^{*} ,$$

36 $$\mathop Q\limits^{\prime } = \mathop \smallint \limits_{0}^{h} r^{*} w^{*} dr^{*} + c\mathop \smallint \limits_{0}^{h} r^{*} cdr^{*} ,$$

37 $$\mathop Q\limits^{\prime } = q + c\frac{{h^{2} }}{2}$$

38 $$\overline{Q} = \frac{1}{T}\mathop \smallint \limits_{0}^{T} \mathop Q\limits^{\prime } dT$$

The dimensionless time-averaged mean flow rate in the laboratory and moving frame is defined as

$$Q = \frac{{\overline{Q}}}{cd}, \;\;\;\; {\text{and}}\;\;\;\; F = \frac{q}{cd}$$

So we get

$$Q = F + \frac{1}{2}\left( {1 + \frac{{\upvarepsilon ^{2} }}{2}} \right),$$

in which $Q$ is a time-mean flow rate calculated for a single period of the wave.

### Solution methodology

The linearized Poisson-Boltzmann solution is solved analytically and the resulting expression for electric potential is utilized in the momentum equation. The simplified set of governing equations is defined in Eqs. (24)–(26) is highly nonlinear and cannot be executed for the analytical solutions. Therefore, the system of governing equations along with boundary conditions given in Eq. (33) is numerically simulated using suitable symbolic computer software i.e. Maple 17 and Mathematica. Furthermore, the graphical results for the flow properties such as temperature, velocity, Nusselt number, and streamlines are computed through Maple 17 to highlight the impact of various physical parameters of the interest.

## Analysis and discussion of results

The main purpose of this section is to outline the impact of the various physical factors on the flow phenomenon of propylene-based titanium dioxide nanofluid through graphical results. The nanoparticles of an average diameter of about 10 nm are dispersed at an initial temperature of 303 K. The fluid flow pattern is visualized by plotting the contour graphs for stream function. Graphical results are prepared by choosing the direction of the applied electric field in such a way that assists the flow of the fluid generated by the beating of the cilia. However, the effect of removing the electric field and reversing the direction of the electric field is also observed. From the mathematical point of view, negative values of the electroosmotic velocity parameter represent the forwarding direction electric field and positive values characterize the opposing electric field. A volume fraction of up to 0.06 vol% of titanium dioxide nanoparticles is scattered in the PG + water solution. The thermophysical attributes of the base solution and nanoparticles are calculated at 303 K and are listed in Table 1. Based on the numerical values given in Table 1, the value of the Prandtl number is found to be 45.078.

**Table 1:** Thermophysical properties of 50:50 PG + water solution and TiO_2_^49^ nanoparticles.

Physical properties	TiO_2_	50:50 PG + water solution
Specific heat $\left( C \right)$	686.2 J/kg K	3570 J/kg K
Density ($\rho$)	4250 kg/m^3^	1038 kg/m^3^
Thermal conductivity $K$	8.9538 W/mK	0.347 W/mK
Particle diameter $\left( d \right)$	10 nm	–
${\text{Dynamic}} {\text{vis}}\cos {\text{ity}}\;\upmu$	–	4.41 × 10^–3^ Pa s

### Analysis of results

**Figure 2:**
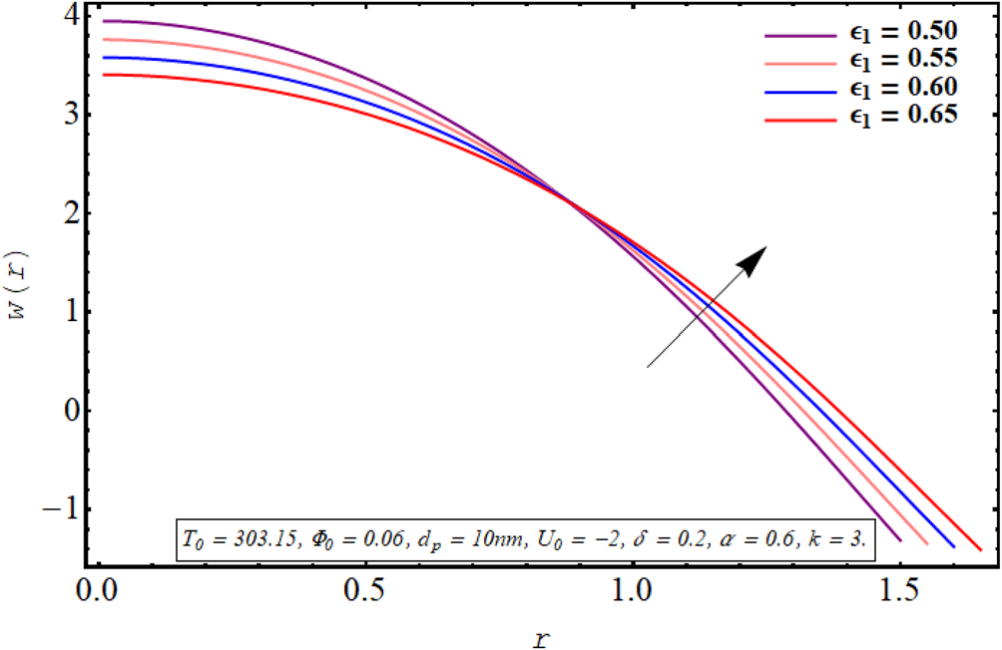
Axial velocity for cilia length parameter.

**Figure 3:**
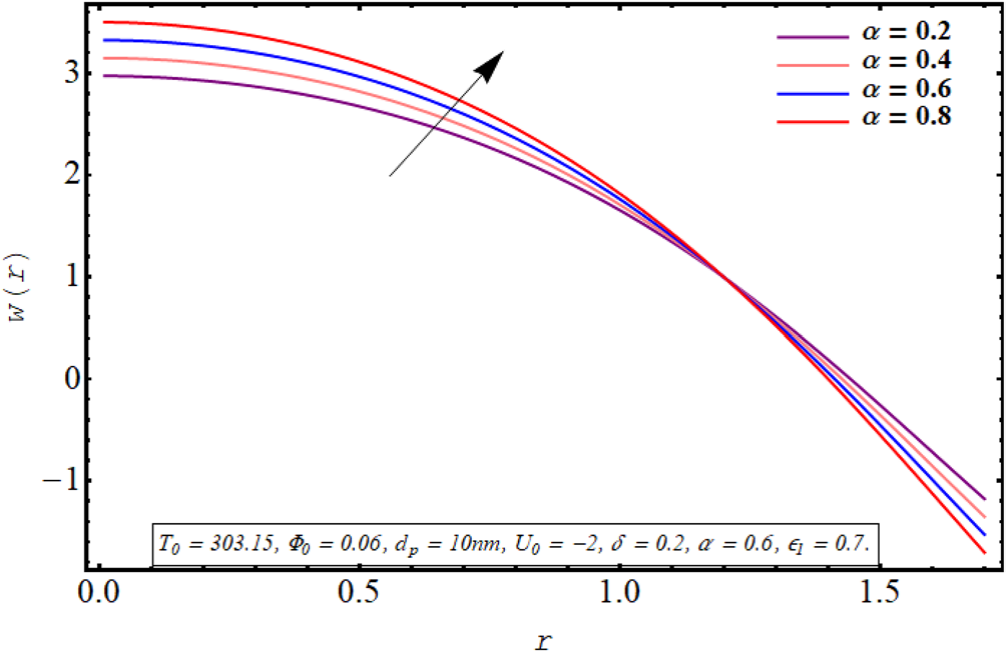
Axial velocity for the eccentricity parameter.

**Figure 4:**
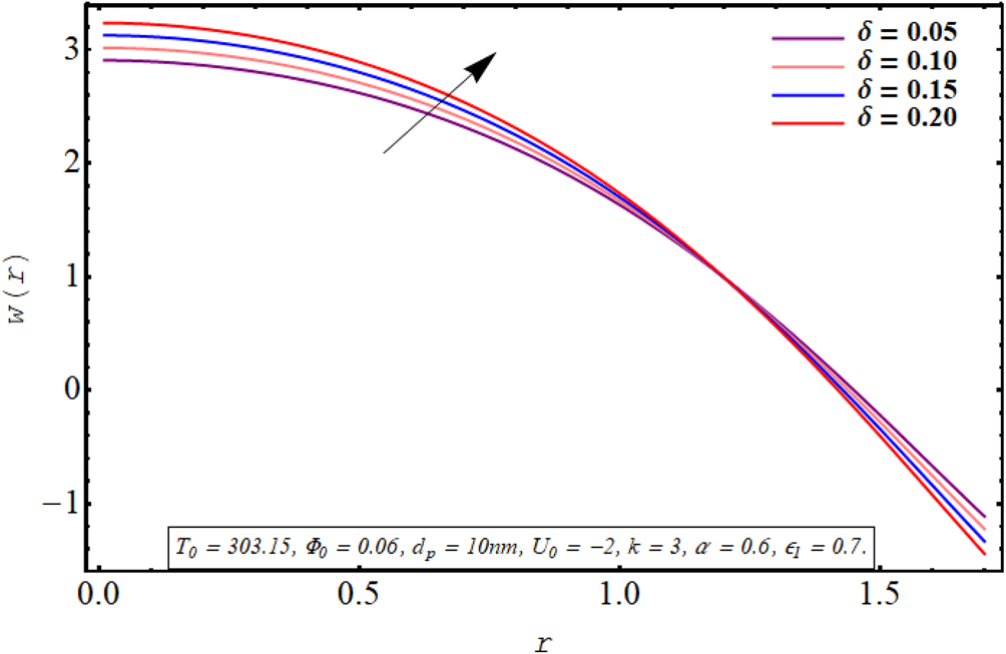
Axial velocity for the wavenumber.

**Figure 5:**
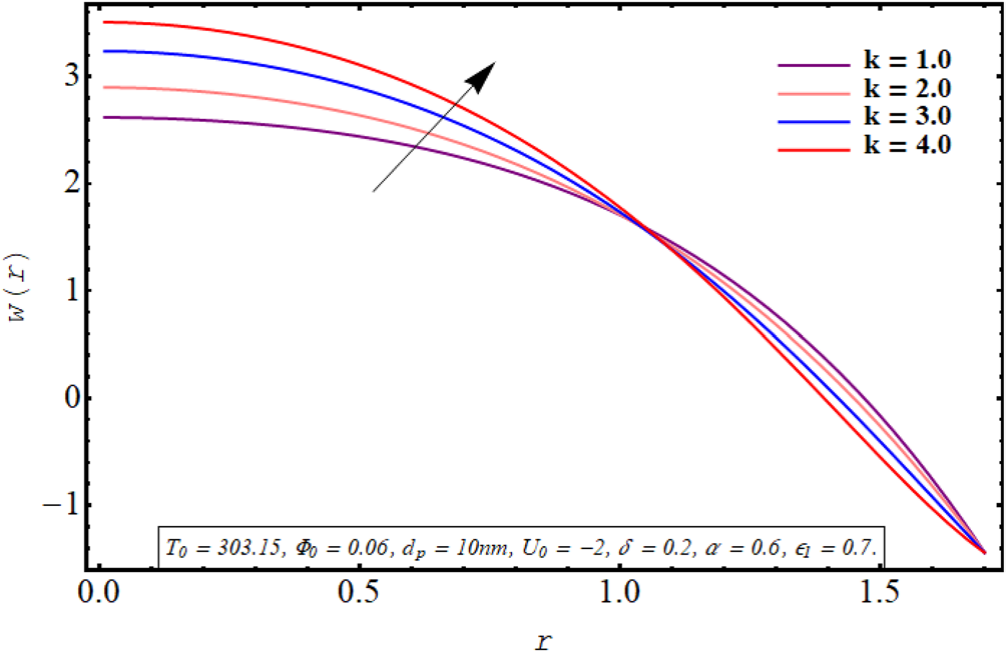
Axial velocity for the Debye length parameter.

**Figure 6:**
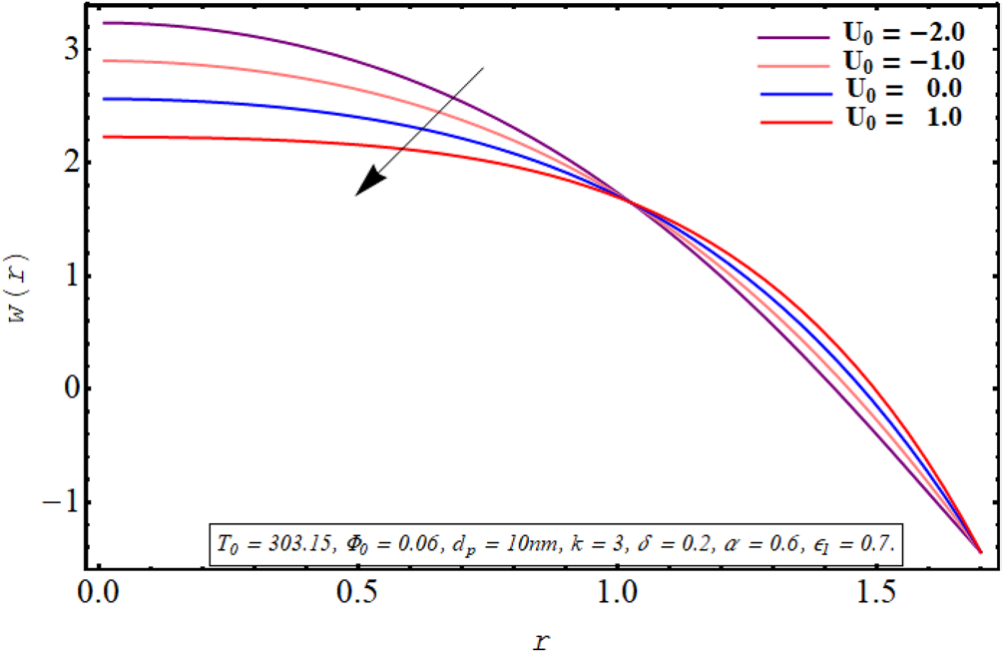
Axial velocity for electroosmotic velocity parameter.

**Figure 7:**
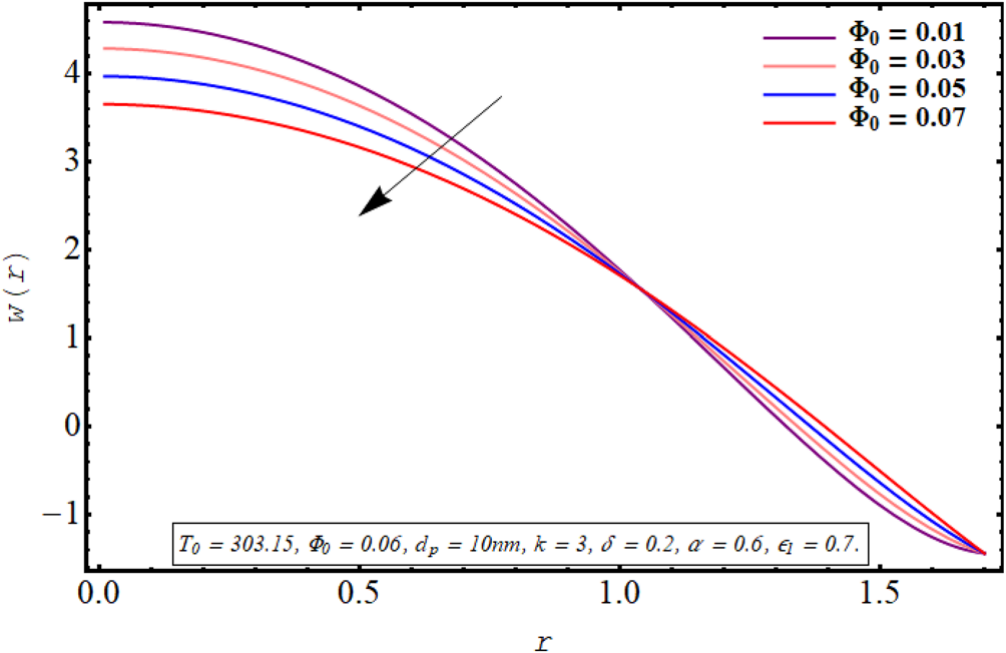
Axial velocity for the nanoparticle volume fraction.

This subsection is devoted to facilitating an insight into the influence of various involved on the velocity distribution of PG + water solution base titanium dioxide nanofluid. The effect of the cilia length parameter on the fluid velocity is characterized in Fig. 2. It can be found that for increasing the length of the cilia in the range of $0.5 \le \epsilon_{1} \le 0.65$, the velocity of the fluid decreases for $0 \le r \le 0.8$ and it increases in the region defined by $0.8 \le r \le 1.5$. The effect of an increase in the eccentricity of the path followed by moving cilia the on velocity profile is analyzed in Fig. 3. Here a rise in the velocity is observed in the central region of the pipe. A noticeable uplift in the fluid motion is produced with a rise in the wave number in the range of $0.05 \le \delta \le 0.20$ as seen in Fig. 4. Figure 5 delineates the impression of the Debye length parameter on the flow phenomenon. A significant rise in the velocity of the fluid in the central region is observed. Another important physical parameter involved in the investigation of the electroosmotic flow phenomenon is the electroosmotic velocity parameter whose effect on fluid velocity is analyzed in Fig. 6. Here the graphical results are being drawn for $U_{0} > 0$, $U_{0,} < 0$ and $U_{0} = 0$ and it is found that velocity is maximum for $U_{0} < 0$ and it is minimal for $U_{0} > 0$. The impact of variation in the fraction of dispersed titanium dioxide nanoparticles on velocity under the same physical conditions is depicted in Fig. 7 and a decline in velocity profile is observed in the resulting panel.

**Figure 8:**
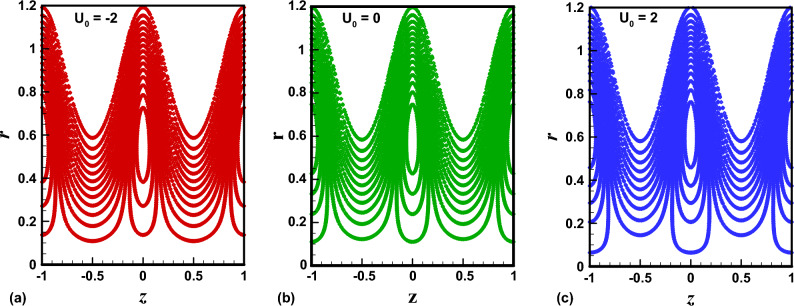
(**a**)–(**c**): Streamline patterns of TiO_2_/PG + water nanofluid for electroosmotic velocity parameter.

**Figure 9:**
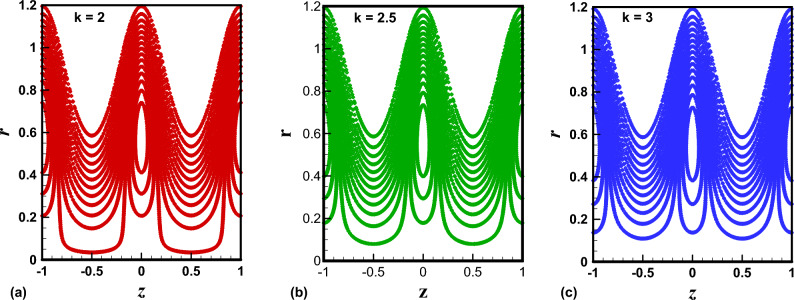
(**a**)–(**c**): Streamline patterns of TiO_2_/PG + water nanofluid for the Debye length parameter.

**Figure 10:**
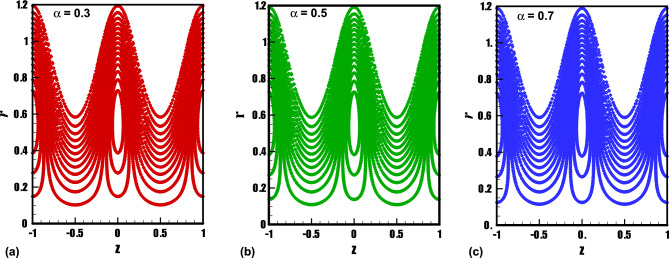
(**a**)–(**c**): Streamline patterns of TiO_2_/PG + water nanofluid for the eccentricity parameter.

**Figure 11:**
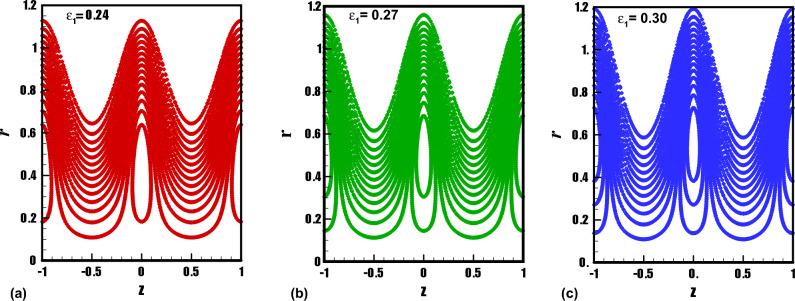
(**a**)–(**c**): Streamline patterns of TiO_2_/PG + water nanofluid for cilia length parameter.

The trapping phenomenon is one of the key features associated with the flows following the pattern of peristaltic pumping. In this process, few streamlines under special circumstances enclose a quantity of the fluid known as trapped bolus which is carried along with the metachronal wave. The area enclosed by the trapping bolus and its shape is mainly affected by involved physical parameters. Therefore, in Figs. 8, 9, 10, 11, contour graphs are plotted for multiple values of the electroosmotic velocity parameter, Debye length parameter, eccentricity, and cilia length parameter to visualize their impact on the circulatory flow pattern. Figure 8a–c illustrate the effect of the electroosmotic velocity parameter on the trapping process. Evidently, with the inclusion of the external electric field in the direction of the main flow direction, less volume of the fluid is trapped when compared with the case of the absence of electric field and the reversal of the electric field direction. Furthermore, the number of closed streamlines is maximum for opposing electric fields and minimum for assisting the electric field. The evolution in the circulatory flow pattern for the rise in the Debye length parameter is depicted in Fig. 9a–c. It can be clearly seen from the resulting panels that there is an increase in the volume occupied by the closed streamlines retaining the same number of closed streamlines in each case. The variation in the size of the trapping bolus via a larger eccentricity parameter is revealed through Fig. 10a–c. Minor growth in the size of the trapped bolus is observed for a rise in the eccentricity of the path followed by the beating of the cilia. Clearly, longer cilia trace a bigger ellipse and the fluid particles attached to these cilia enclose a comparatively larger fluid bolus. A prominent growth in the circulatory flow pattern is observed for enhancement in the characteristic length of the cilia as manifested in Fig. 11a–c. Thus it can be concluded that the length of the cilia generating the metachronal wave is the most significant parameter affecting the fluid flow dynamics.

**Figure 12:**
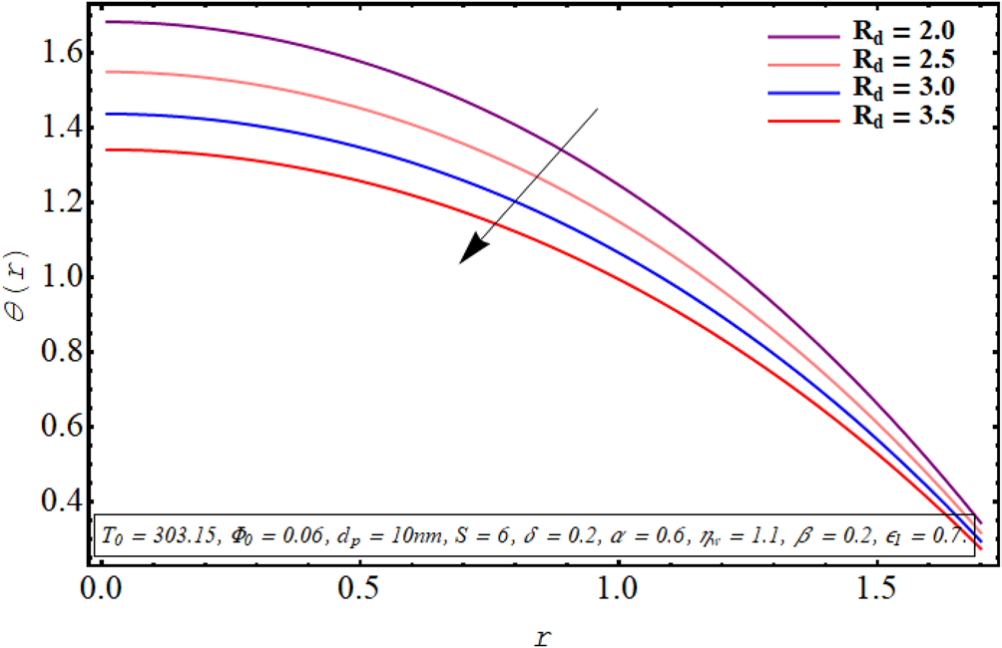
Temperature for the radiation parameter.

**Figure 13:**
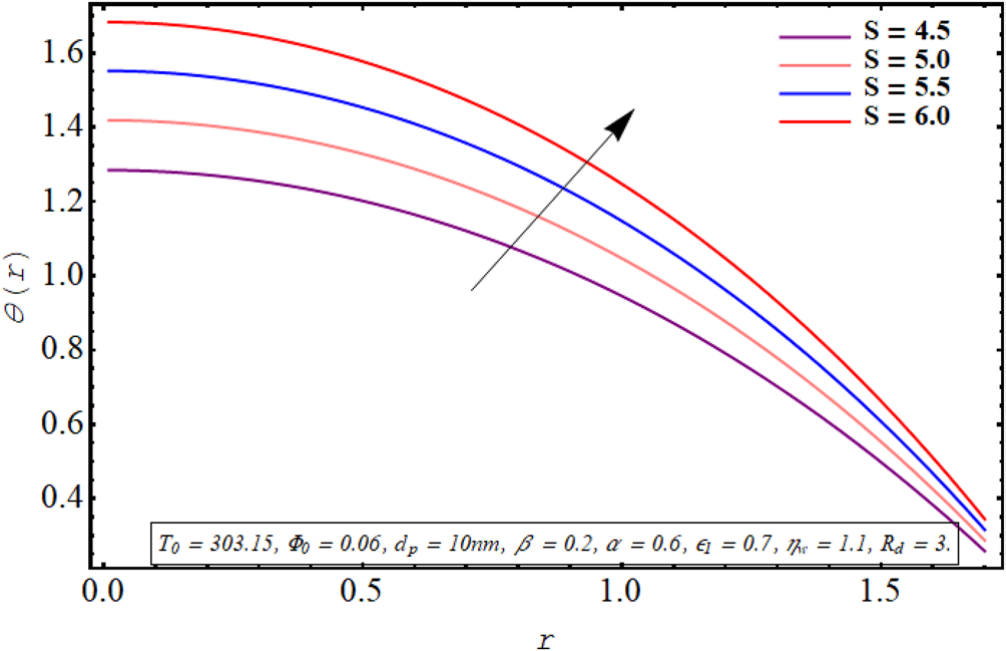
Temperature for the Joule heating parameter.

**Figure 14:**
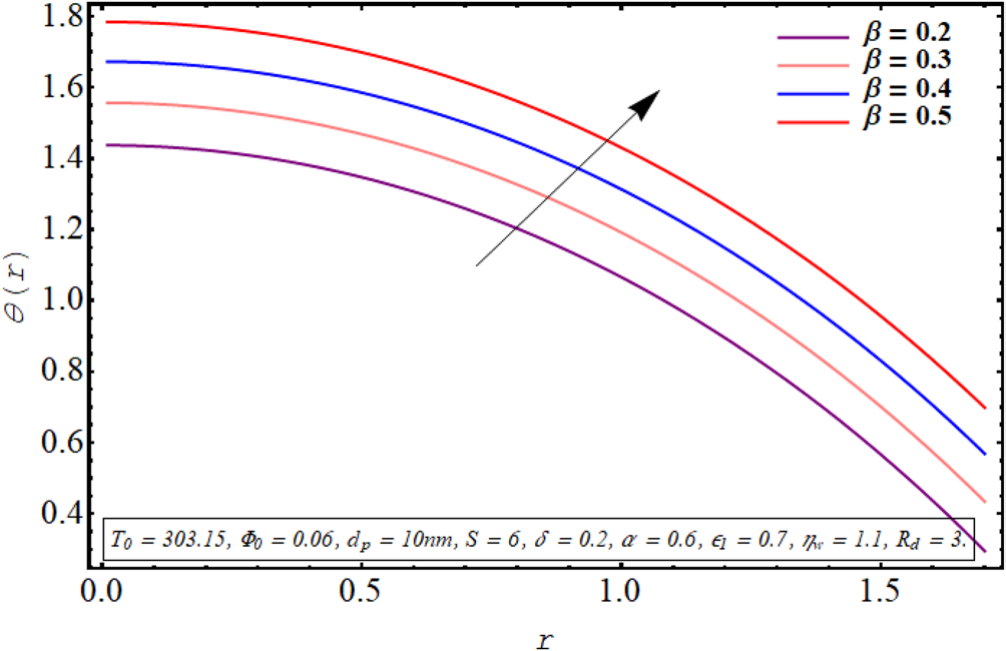
Temperature profile for thermal slip parameter.

**Figure 15:**
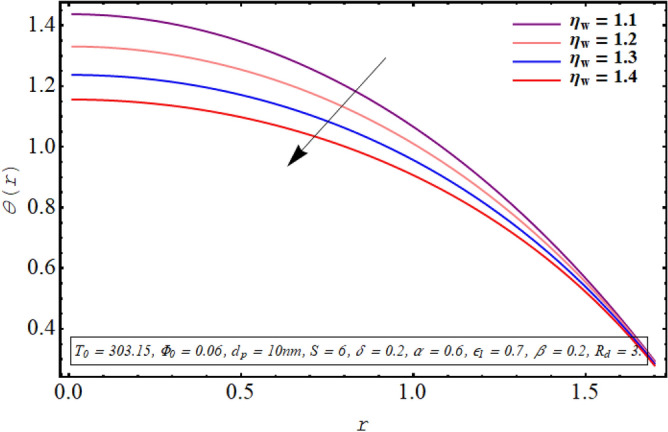
Temperature profile for the temperature ratio parameter.

**Figure 16:**
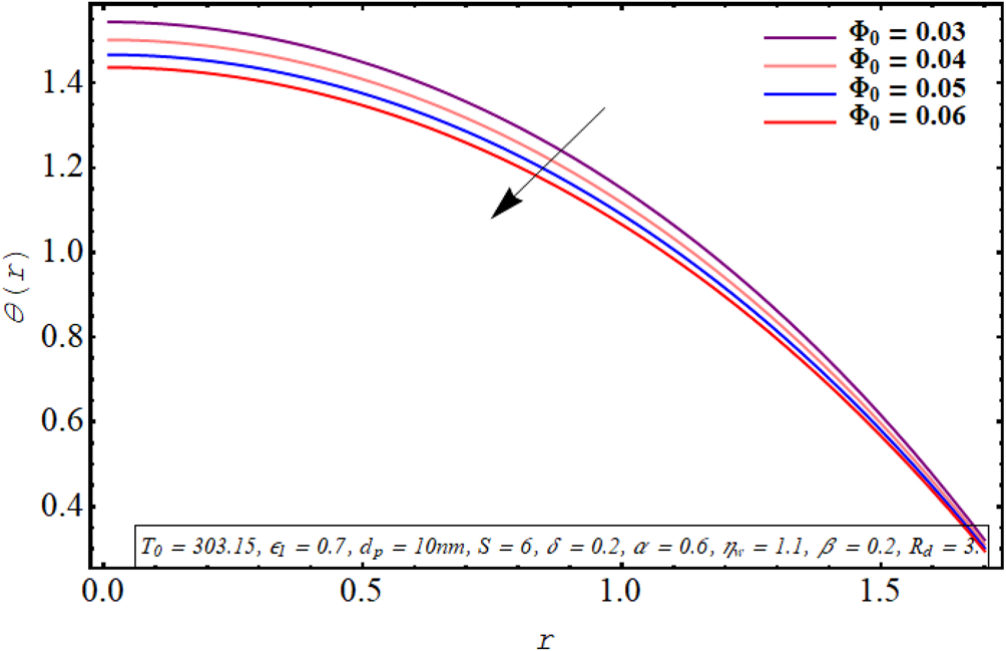
Temperature for nanoparticle volume fraction.

**Figure 17:**
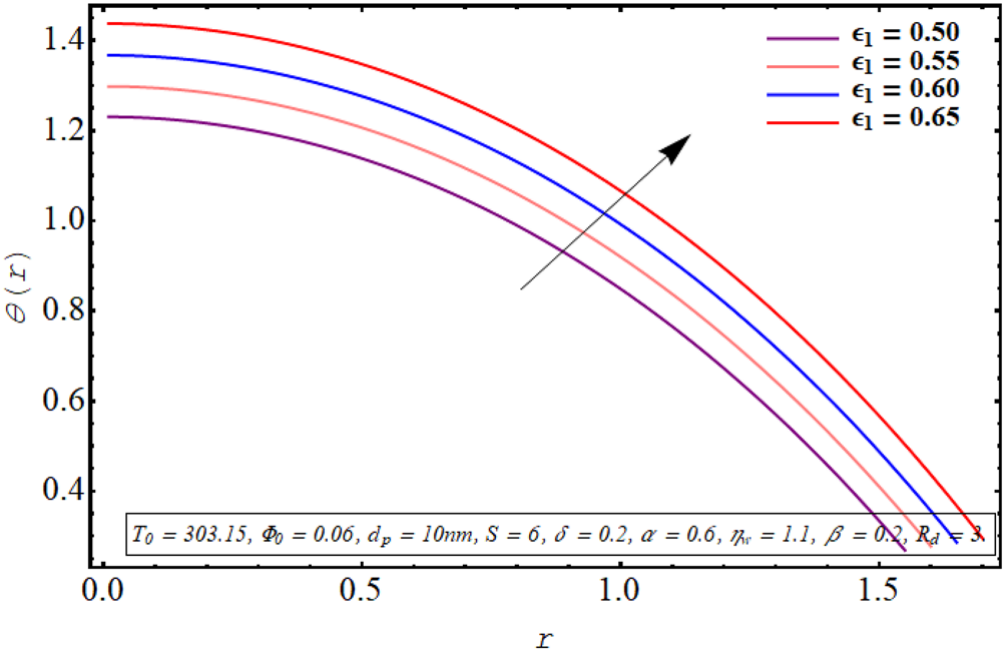
Temperature for the cilia length parameter.

The alteration in the temperature subject to variation in different embedded parameters is displayed in Figs. 12, 13, 14, 15, 16, 17. The modification in the temperature profile for rising values of the radiation parameter from 2.0 to 3.5 is indicated in Fig. 12. A remarkable reduction in temperature profiles is observed for increment in the thermal radiation parameter. The development in the temperature distribution for multiple values Joule heating parameter in the range of $4.5 \le S \le 6.0$ is analyzed in Fig. 13. It is clear from the resulting sketch that there is a rise in temperature of the nanofluid subject to a rise in S. The impact of the thermal slip parameter on temperature distribution is elaborated in Fig. 14. Here a consistent augmentation in the temperature of the fluid is observed. Figure 15 is plotted to analyze the variation in the thermal distribution for higher values of the temperature ratio parameter from $1.1 \le \eta_{w} \le 1.4$. A substantial suppression in the temperature profile is depicted in the resulting graph. Figure 16 portends a significant drop in the temperature of the fluid for increment in the nanoparticle volume fraction from 0.03 to 0.06 in the base fluid. Figure 17 is prepared to examine the influence of the cilia length parameter on temperature. The temperature of the nanofluid is elevated when longer cilia are used for generating the metachronal wave.

The non-dimensional Nusselt number is expressed as:

35 $${\text{Nu}} = - \frac{{ k_{nf} }}{{k_{bf} }}\frac{\partial \theta }{{\partial r}}|_{r\rightarrow h}.$$

**Figure 18:**
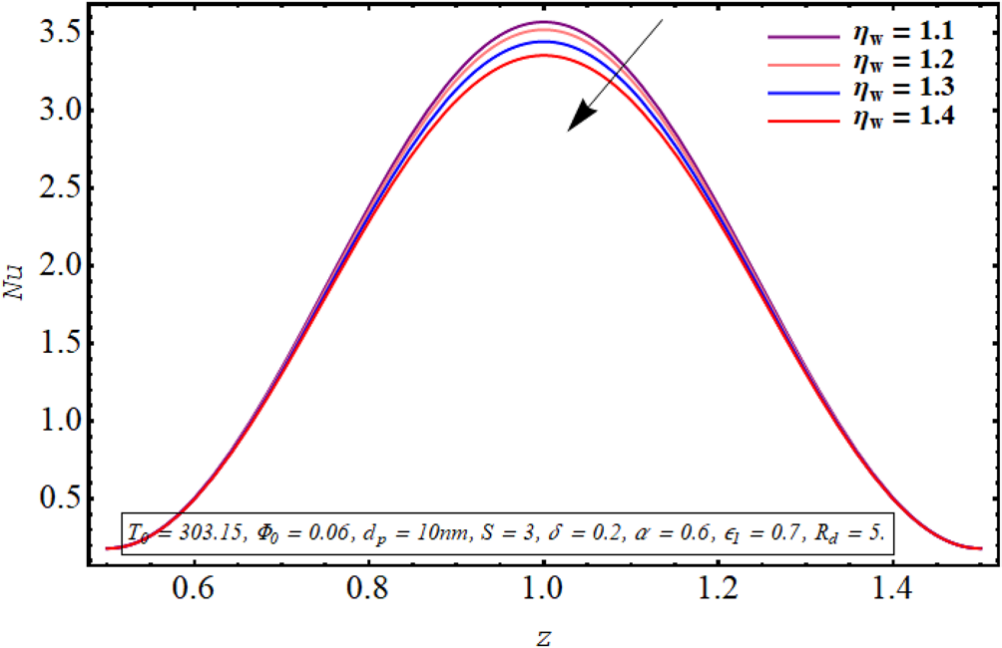
Nusselt number for temperature ratio parameter.

**Figure 19:**
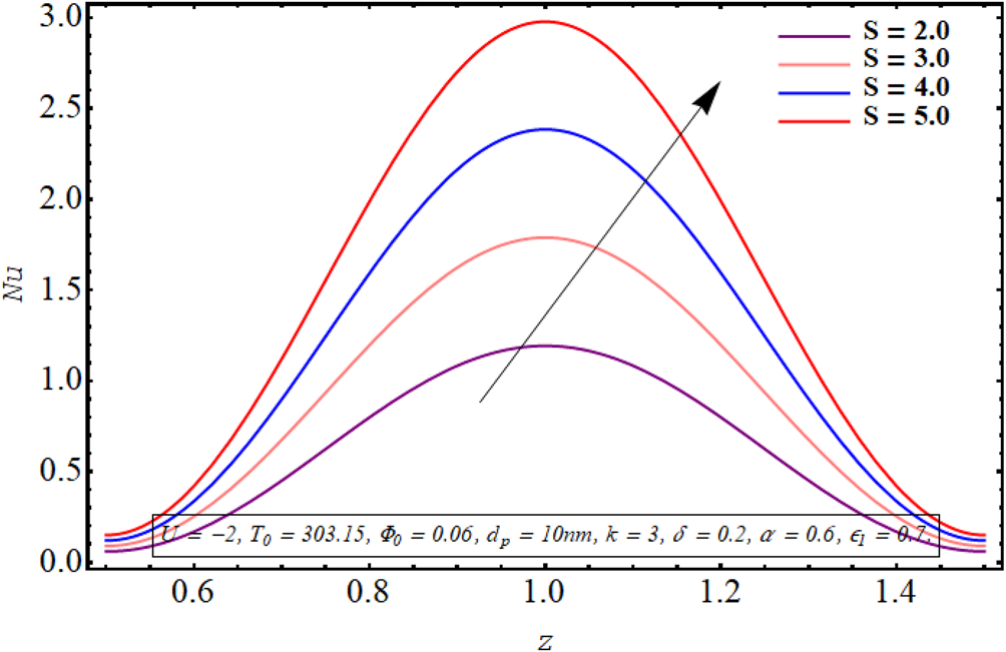
Nusselt number for the Joule heating parameter.

**Figure 20:**
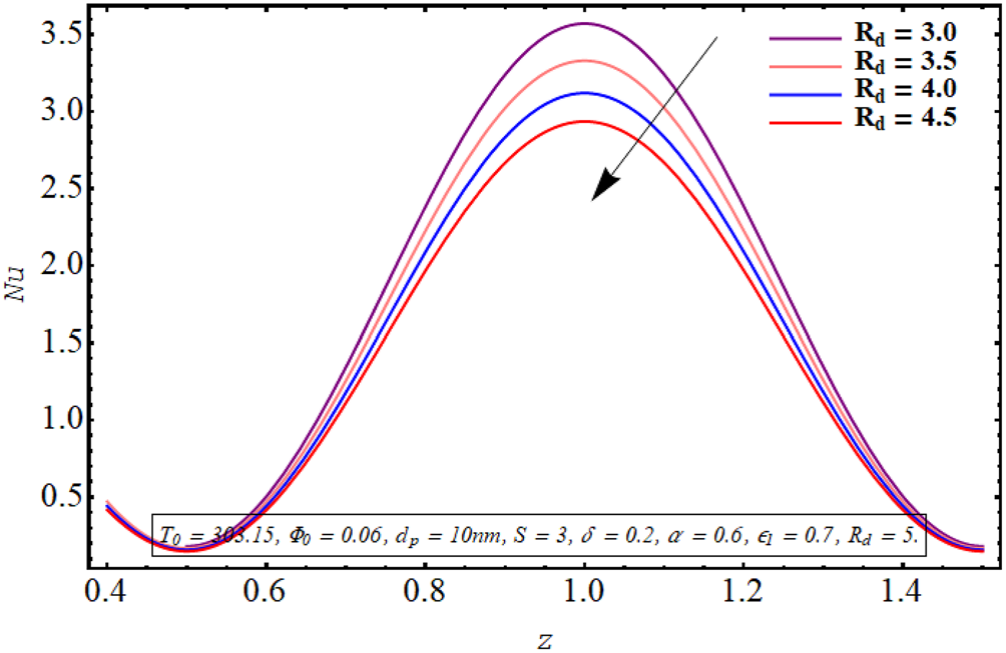
Nusselt number for radiation parameter.

**Figure 21:**
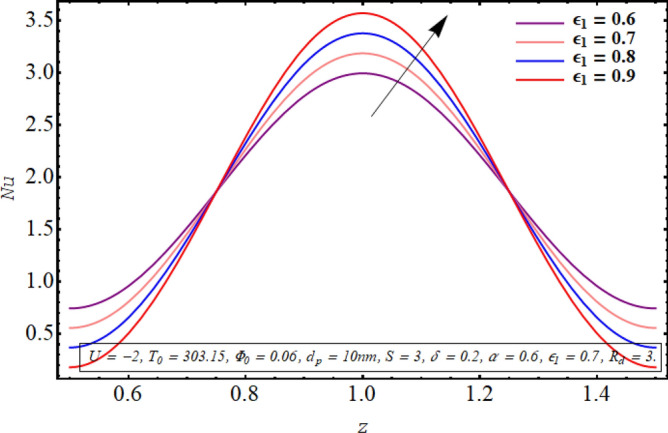
Nusselt number for the cilia length parameter.

**Figure 22:**
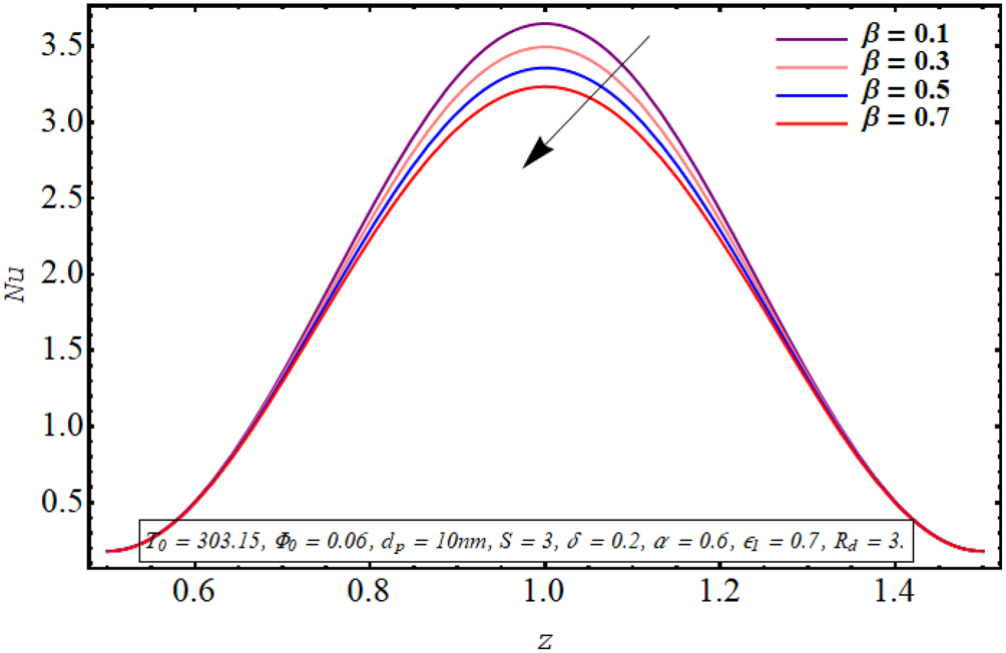
Nusselt number for the thermal slip parameter.

Figures 18, 19, 20, 21, 22 are sketched to observe the characteristics of the Nusselt number for variation in different involved parameters. Nusselt number is a parameter that facilitates measuring the heat transfer ability of the working fluid. The variation in the distribution of the Nusselt number along the micro tube walls for rising values of the temperature ratio parameter is assessed in Fig. 18. One may note that there is a depression in the magnitude of the Nusselt number when the temperature ratio is increased. Nusselt number distribution for multiple values of the Joule hearing parameter can be visualized in Fig. 19. The change in Nusselt number for rising values of the thermal radiation parameter is depicted in Fig. 20. The resulting graph reveals a decline in Nusselt number. It is noticed from Fig. 21 that for increasing the length of the cilia involved in generating the fluid flow, Nusselt number declines. The impact of the temperature slip parameter on the Nusselt number along the tube wall is elucidated in Fig. 22. An intensification is observed in the Nusselt number when more thermal slip is experienced by the fluid at microtube walls.

## Discussion of the results

Physically a rise in the length of the cilia corresponds to an increase in the amplitude of the metachronal wave as well as in its wavelength, therefore, the fluid velocity is boosted for larger $\epsilon_{1}$ as observed through Fig. 2. The reason behind the increment in velocity profile for increase in eccentricity parameter can be related to the fact that eccentricity parameter is directly proportional to the cilia length which causes a rise in the driving force generated by the beating of the cilia. The result obtained in Fig. 4 for larger wave number can be validated by the fact that when the wavenumber is increased, a dominance of inertial forces over viscous forces is produced which facilitates the fluid motion. Basically, the Debye length parameter is inversely related to the thickness of the electric double layer and a thin EDL corresponds to the uneven distribution of electric potential within the fluid medium. Consequently, the electroosmotic fluid flow is augmented and a rise in axial velocity is observed as depicted in Fig. 5. The Debye length parameter is one of the most important parameters in the electro-osmotically modulated flows and it can be visualized that it is a very influential parameter that is very helpful in controlling the speed of the fluid. As already mentioned that negative values of electroosmotic velocity represent that the axial electric field is oriented in the positive z-direction which physically means that in this case, the electroosmotic velocity is in the direction of the fluid flow generated by the motion of cilia and a positive value means that electroosmotic process is occurring is the backward direction opposite to main flow direction, therefore, it can be observed from Fig. 6 that velocity of the nanofluid is maximum for a negative value of $U_{0}$ and it is minimum for positive values. However, $U_{0} = 0$ physically corresponds to the absence of the external electric field and in this case, fluid motion is occurring only due to ciliary motion, therefore, the velocity profile lies in between the above-mentioned cases of assisting and resisting electric field. As the viscosity of the base fluid rises when the concentration of nanoparticles is enhanced which in turn resists the fluid flow, and a decline in velocity profile is observed in Fig. 7. In all of the Figs. 2, 3, 4, 5, 6, 7, an opposite response of the velocity profile is observed near the wall of the channel, which can be justified by the fact that in order to maintain the fixed flow rate, velocity of the fluid depicts the opposite trends.

For growth in the radiation parameter, the process of conductive heat transfer from the fluid medium is boosted which in turn reduces the temperature profile (See Fig. 12). The Joule heating phenomenon measure the resistance offered by electrolyte solution to the passage of current. A larger Joule heating parameter quantifies the larger amount of resistance experienced by electric current. As a result, more electric energy is converted into the heat energy and temperature of the fluid increases as observed in Fig. 13. As $\eta_{w}$ is the ratio of temperature at the upper wall and at the lower wall, so larger $\eta_{w}$ corresponds to an elevation in temperature difference which boosts the heat transfer rate within the fluid medium and the overall temperature of the fluid drops as noticed through Fig. 15. A keen examination of thermal conductivity relation for nanofluid given in Eq. (15) clarifies that the thermal conductivity of the nanofluid is directly influenced by the fraction of nanoparticles being suspended in the base fluid which justifies its effect of boosting the cooling phenomenon and decreasing the fluid temperature in Fig. 16. These enhanced thermal properties of the fluid boost the efficiency of the working fluid by helping in controlling the fluid temperature. When the cilia inside the tube have a relatively larger length, their movement causes a stronger pushing force on the fluid which causes the temperature of the fluid to increase as shown in Fig. 17.

Nusselt number quantifies the relative measure of heat transfer by convection as compared to conductive heat transfer. It can be concluded from Figs. 18 and 20 that for a larger temperature difference which is related with temperature ratio can boost the other modes of heat transfer such as heat transfer by radiation, heat transfer associated with Brownian and thermophoretic diffusion of nanoparticles, therefore a reduction is convective rate of heat transfer occurs and Nusselt number declines. Moreover, as Joule heating parameter ${\text{S}} = \frac{{\sigma_{bf} U_{{E_{z} }}^{2} d^{2} }}{{k_{bf} (T_{1} - T_{0} )}}$ is strongly dependent on the strength of applied electric field, which is the source of fluid motion in the forwarding direction in case of negative values of $U_{0}$, therefore larger values of S raises the convective heat transfer and the Nusselt number increases significantly (See Fig. 19). The result in Fig. 21 can be justified by the fact that using relatively larger cilia facilitates the movement of fluid which raises the heat transfer due to convection. A larger temperature jump parameter quantifies larger temperature difference between the fluid and the solid surface, which corresponds to larger kinetic energy of the fluid particles. As a result, momentum diffusivity increases and Nusselt number also raises due to rapid movement of heated fluid as observed in Fig. 22.

## Concluding remarks

Herein, a theoretical investigation has been conducted to study the heat transfer characteristics of 50:50 PG + water -based ionic solution whose motion is driven by the combined effects of electroosmotic body forces and the beating of the cilia. In presence of nonlinear thermal radiation and Joule heating, a nonlinear coupled system of governing equations is simulated by mathematical software Maple 17. The significant findings of the current analysis are listed as:

- Propylene glycol and water-based Titania nanofluid is the most suitable heat transfer fluid in the household solar device which enhances heat transfer capability with the reduction of fluid temperature.
- The application of the electric field along the positive axial direction boosts the fluid velocity which results in the rapid removal of the heat from the system.
- The length of cilia involved in generating the fluid motion strongly influence the velocity, temperature, and trapping phenomenon and it also boosts the heat transfer tendency of the fluid
- Increasing the thermal radiation parameter results in the reduction of fluid temperature.

## Data Availability

The datasets used and/or analysed during the current study available from the corresponding author on reasonable request.

## Abbreviations

$d$: The half-width of the channel ${\text{m}}$
$${\upeta }$$: Wavelength $${\text{m}}$$
$\tilde{r}, \tilde{z}$: Radial and axial coodinates ${\text{m}}$
$\tilde{u} ,\tilde{w}$: Radial and axial velocity component ${\text{m}}\,{\text{s}}^{ - 1}$
$\rho_{nf} ,\rho_{fb} ,\rho_{p}$: The nanofluid, base fluid, and nanoparticles density respectively ${\text{Kg}}\,{\text{m}}^{ - 3}$
$$\tilde{t}$$: Time scale $${\text{s}}$$
$$\tilde{p}$$: Pressure force $${\text{N}}\,{\text{m}}^{ - 2}$$
$\rho_{e}$: Local electric charge density ${\text{C}}\,{\text{m}}^{ - 3}$
$\gamma_{nf} ,\gamma_{s} ,\gamma_{bf}$: Thermal expansion coefficient for nanofluid, solid particles and basefluid ${\text{K}}^{ - 1}$
$U_{{E_{{\tilde{r}}} }} ,U_{{E_{{\tilde{z}}} }}$: The radial and axial electric body field ${\text{Kg}}\,{\text{ms}}^{ - 3} \,{\text{A}}^{ - 1}$
$C_{nf} , C_{p} ,C_{bf}$: Specific heat of nanofluid and nanoparticles ${\text{J}}\,{\text{Kg}}^{ - 1} \,{\text{K}}^{ - 1}$
$$\tilde{T}$$: Temperature field $${\text{K}}$$
$D_{B}$: Brownian diffusion parameter ${\text{m}}^{2} \,{\text{s}}^{ - 1}$
$$\tilde{\Phi }$$: Concentration field $${\text{Kg}}\,{\text{m}}^{ - 3}$$
$\sigma_{nf} ,\sigma_{bf} ,\sigma_{p}$: Electrical conductivity of the nanofluid, base fluid, and nanoparticles. ${\text{S}}\,{\text{m}}^{ - 1}$
$$D_{{\tilde{T}}}$$: Thermophoretic parameter $${\text{m}}^{2} \,{\text{s}}^{ - 1}$$
$k_{nf} ,k_{bf} ,k_{p}$: Nanofluid, basefluid, and nanoparticle thermal conductivity. ${\text{W}}\,{\text{m}}^{ - 1} \,{\text{K}}^{ - 1}$
$d_{p}$: The diameter of nanoparticles ${\text{m}}$
$\sigma^{*}$: Stefan-Boltzmann constant ${\text{W}}\,{\text{m}}^{ - 2} \,{\text{K}}^{ - 4}$
$\kappa^{*}$: Mean absorption coefficient ${\text{m}}^{ - 1}$
$T_{fr}$: The freezing temperature of the base fluid ${\text{K}}$
$$U_{{\tilde{E}}}$$: Electric potential $${\text{V}}$$
$\varepsilon_{0}$: Vacuum’s dielectric constant ${\text{Fm}}^{ - 1}$
$n_{0}$: The bulk concentration of ionic species ${\text{Kg}}\,{\text{m}}^{ - 3}$
$e$: Charge of electron ${\text{C}}$
$\Phi_{0}$: Nanoparticle volume fraction [–]
$\varepsilon_{r}$: Fluid’s relative permittivity [–]
$$z$$: Valence [–]
$p$: Dimensionless pressure parameter [–]
$u,w$: Dimensionless velocity components [–]
$k$: Debye length parameter [–]
$$U_{0}$$: Electroosmotic velocity [–]
$N_{t}$: Dimensionless thermophoretic parameter [–]
${\text{Gr}}_{\Phi }$: Mass Grashof number [–]
$R_{d}$: Dimensionless radiation parameter [–]
$\eta_{w}$: Temperature ratio parameter [–]
$N_{b}$: Dimensionless Brownian diffusion parameter [–]
$\Psi$: Dimensionless stream function [–]
$S$: Joule heating parameter [–]
${\text{Gr}}_{{\text{t}}}$: Temprature Grashof number [–]
$${\text{Pr}}$$: Prandtl number [–]
$$\delta$$: Wave number [–]
${\text{Re}}$: The Reynolds number [–]
$\beta$: Thermal slip parameter [–]
$\xi$: Dimensionless zeta potential [–]
$${\text{Nu}}$$: Nusselt number [–]
$Q$: The dimensionless averaged flux [–]
θ: Dimensionless temperature [–]
Φ: The dimensionless nanoparticle volume fraction [–]
